# Nutritional Contribution and Bioactive Properties of Romanian Monoecious Hemp Seed (*Cannabis sativa* L.) Varieties for Potential Food Applications

**DOI:** 10.3390/foods15193363

**Published:** 2026-09-22

**Authors:** Petru Alexandru Vlaicu, Arabela Elena Untea, Iulia Varzaru, Lorena-Diana Popa, Raluca Paula Turcu, Mihaela Saracila, Florin Nenciu

**Affiliations:** 1 Feed and Food Quality Department, National Research and Development Institute for Animal Biology and Nutrition, Calea București No. 1, 077015 Balotesti, Ilfov, Romania; arabela.untea@ibna.ro (A.E.U.); iulia.maros@ibna.ro (I.V.); raluca.turcu@ibna.ro (R.P.T.); mihaela.saracila@ibna.ro (M.S.); 2 Agricultural Research and Development Station Secuieni, Agriculturii Street No. 15, 617415 Secuieni, Neamt, Romania; 3 Testing Department, National Institute of Research-Development for Machines and Installations Designed to Agriculture and Food Industry—INMA Bucharest, 6 Ion Ionescu de la Brad Avenue, 013813 Bucharest, Romania; florin.nenciu@inma.ro

**Keywords:** hemp seed, nutritional profile, Romanian varieties, food, nutrients, food ingredients

## Abstract

Hemp seeds (*Cannabis sativa* L.) are nutrient-dense, plant-based food ingredients. However, cultivar-related variability in its compositional profile remains insufficiently characterized for Romanian varieties. This study evaluated six Romanian monoecious hemp seed varieties (Dacia Secuieni, Secuieni Jubileu, Ratza, Zenit, Succesiv, and Olivia), for their potential food applications. Dacia Secuieni, Secuieni Jubileu, Ratza, and Olivia showed the highest crude protein content (24.79–25.57%). Secuieni Jubileu contained the highest crude fat (30.96%) and α-tocopherol (31.14 mg/kg), with the lowest total phenolic content (0.74 mg GAE/g) and carotenoids. Dacia Secuieni had the highest phosphorus (1.32%), manganese (138.91 mg/kg), total tocopherol (227.44 mg/kg), and α-linolenic acid concentration (19.76% of total FA), with an adequate omega-6/omega-3 ratio (2.78). Ratza was characterized by the highest crude fiber content (38.85%), oleic acid (16.47%), and canthaxanthin content (1.10 mg/kg), and it exhibited the highest omega-6/omega-3 ratio (3.64%). Zenit contained the highest iron (105.21 mg/kg) and γ-linolenic acid concentration (4.307%), was second for crude fiber (37.97%), and displayed the strongest hydrosoluble antioxidant activity, total phenolic content (1.50 mg GAE/g), and ABTS radical-scavenging activity (14.97 mmol TE/kg dw). Succesiv had the lowest crude protein (23.96%) and fat (25.62%) content, but it had the highest lutein content (79.46 mg/kg), DPPH radical-scavenging activity (12.89 mmol TE/kg dw), and metal-chelating activity (3.15 mg/g EDTA), while it was second in terms of linoleic acid proportion (56.11%) and γ-linolenic acid concentration (4.116%). Olivia exhibited the highest zinc content (90.35 mg/kg), linoleic acid proportion (56.16%), and total antioxidant capacity (49.70 mmol AAE/kg dw). These results indicate that Romanian hemp seed varieties are valuable sources of protein, essential fatty acids, minerals, and antioxidant compounds. The observed variability supports cultivar-specific selection for the development of hemp-based functional food ingredients.

## 1. Introduction

Industrial hemp (*Cannabis sativa* L.) is among the oldest cultivated crops worldwide and has recently experienced renewed interest due to its nutritional value, sustainability, and versatility in food applications [1,2]. Unlike drug-type, high-THC cannabis varieties (*Cannabis indica*), industrial hemp varieties cultivated within the European Union contain low concentrations of Δ9-tetrahydrocannabinol (THC) and are legally authorized for agricultural production under specific regulatory frameworks [3]. Following the revision of the Common Agricultural Policy, hemp varieties eligible for cultivation in the European Union must contain less than 0.3% THC. The Commission Regulation (EU) 2022/1393 first established maximum levels for THC in hemp-derived foods (3.0 mg/kg for seeds and 7.5 mg/kg for oil), expressed as Δ9-THC + 0.877 × Δ9-THCA [3]. These limits are now consolidated under Commission Regulation (EU) 2023/915 [4], which repealed Regulation (EC) No. 1881/2006. These legislative developments have stimulated the expansion of hemp-based food products while simultaneously increasing the need for comprehensive compositional characterization of cultivars intended for human consumption.

Current consumer demand for plant-based, minimally processed, and nutritionally dense foods has positioned hemp seeds as one of the most promising alternative ingredients for the food sector [5,6]. Currently, they are used in different food products (edible oils, protein concentrates, plant-based beverages, bakery products, snacks, fermented foods) and formulations [7,8,9,10], due to their favorable nutritional profile, which combines high-quality proteins, essential fatty acids, dietary fibers, minerals, vitamins, and other phytochemicals [11]. The protein content of different hemp seed cultivars ranges between 20% to 30%, depending on cultivar and growing conditions [12,13], having a good digestibility and a balanced amino acid profile [12]. The lipid (25% to 35%) content of hemp seed includes more than 70% polyunsaturated fatty acids (PUFAs), primarily linoleic acid (LA; 18:2n-6) and α-linolenic (ALA; 18:3n-3) fatty acids (FAs) [14,15]. The typical omega-6/omega-3 ratio (3:1) is considered more nutritionally favorable compared with other conventional oilseed crops [14,16]. Beyond their macronutrient composition, hemp seeds also represent a valuable source of bioactive compounds. Previous studies have reported notable concentrations of tocopherols, polyphenols, and flavonoids, but little information about carotenoids. These bioactive compounds contribute significantly to the antioxidant capacity and oxidative stability of hemp seed-derived foods [12,17]. Several studies have reported that the cultivar is one of the major factors affecting hemp seed composition. Vonapartis et al. [17] reported significant cultivar-dependent differences in FA composition and tocopherol concentrations among Canadian hemp cultivars, while Irakli et al. [12] noted significant variability in protein, lipid, mineral, and phenolic contents among some European cultivars. Similar conclusions were reached by Siano et al. [13], highlighting major differences in antioxidant compounds and nutritional quality traits across commercial hemp seed cultivars. These findings highlight the importance of cultivar-specific evaluation when selecting hemp seed for food applications and functional ingredient development.

Romania has a long tradition of hemp cultivation and possesses favorable pedoclimatic conditions for this crop cultivation. Dacia Secuieni is a cultivar primarily developed for stem and fiber production. Secuieni Jubileu is a cultivar mainly selected for seed and oil production. Ratza cultivar was developed primarily for biomass production. Zenit is an established Romanian cultivar intended mainly for seed and oil production, being used as a reference cultivar in comparative hemp evaluations. Succesiv is a cultivar characterized by good agronomic flexibility and suitability for successive cropping systems, combining biomass and seed production. Olivia is a modern dual-purpose cultivar suitable for both seed and fiber production.

Although there is an increasing interest in hemp seed-derived foods, comparative information regarding the nutritional composition of monoecious hemp seeds cultivated under Romanian conditions remains scarce. A recent study by Floareș Oarga et al. [18] characterized six dioecious Romanian hemp varieties (Lovrin 110, Silvana, Armanca, Teodora, and two lines under certification) from the Lovrin research station, comparing conventional and ultrasound-assisted extraction for polyphenol, flavonoid, and DPPH antioxidant activity, without FA, tocopherol, or carotenoid analysis. The present study assessed six monoecious cultivars from the Secuieni research station. It provides a broader compositional dataset including FA profiles (including minor components such as GLA, stearidonic acid, and odd-chain FAs), tocopherols, and carotenoid profiles, not previously reported for Romanian monoecious hemp seed. This study applied PCA to integrate multiple selected representative parameters from the FA, mineral, and bioactive-compound parameters simultaneously. In this context, the objective of the present study was to comparatively characterize six different monoecious hemp seeds (*Cannabis sativa* L.) from Romania through the assessment of proximate composition, mineral content, FA profile, and hydrosoluble and liposoluble antioxidants, with the aim of evaluating their potential utilization as food ingredients and identifying the cultivar with superior nutritional and potential functional properties. We hypothesized that the six monoecious cultivars would differ significantly in nutritional and bioactive composition, reflecting their specific characteristics, and that this variation would support cultivar-specific recommendations for food applications.

## 2. Materials and Methods

### 2.1. Sourcing of Plant Material

Six different cultivars of hemp seeds were supplied by the project partner Statiunea de Cercetare-Dezvoltare Agricola Secuieni, from their own crops, located in Secuieni, Neamt County, Romania (46°51′20″ N 26°50′56″ E). The experimental plots were arranged in three blocks, with each cultivar represented once within each block. The seeds of each cultivar were harvested in 2023 from three independently sampled production plots (biological replicates). The cultivation method, planting soil, climate conditions, sowing (April), and harvesting (August to September, depending on the cultivar maturity stage) are described elsewhere [19]. All cultivars used in this study are included in the Plant Variety Database of the European Commission [20], as low-THC (<0.3%) authorized hemp varieties: “Dacia Secuieni” (EU species code 85; variety ID: 213917), “Secuieni Jubileu” (EU species code 85; variety ID: 214000), “Ratza” (EU species code 85; variety ID: 213995, “Zenit” (EU species code 85; variety ID: 214019), “Succesiv” (EU species code 85; variety ID: 214008), and “Olivia” (EU species code 85; variety ID: 213989). Before analysis, the seeds were dried at 65 °C in an oven for 24–48 h, until constant weight was reached. Then, the samples were ground into a fine powder using a Grindomix GM 200 mill (Retsch, Haan, Germany) with a 0.5 mm sieve. The powders were stored in paper bags at room temperature (18–20 °C), protected from direct light, for approximately two days before analysis. All samples were subjected to the same drying, storage, and milling conditions. Each plot-level sample was subsequently analyzed in triplicate (technical replicates) for every determination reported. Comparisons in this study represent three field plots per cultivar at a single location (Secuieni, Neamt) and harvest year (2023).

### 2.2. Nutritional and Mineral Contents Analyses

The primary chemical composition analysis of the hemp seeds was carried out following the methods recommended by Regulation (CE) nr. 152/2009 and ISO standard authorized techniques [21]. Dry matter (DM) was determined gravimetrically according to ISO 6496:2001 [22] in an Ecocell oven. Crude protein (CP) was determined by the Kjeldahl method (nitrogen-to-protein conversion factor 6.25; Kjeltec auto 1030 Tecator Instruments, Höganäs, Sweden), according to ISO 5983-2:2009 [23]. The crude fat (EE) was determined according to SR ISO 6492:2001 [24], using a Soxhlet apparatus, via extraction in petroleum ether (Soxtec 2055 Foss Tecator, Höganäs, Sweden). The crude fiber (CF) was determined via intermediary filtration, according to SR EN ISO 6865:2002 [25], with a Fibertec 2010 System Foss Tecator, (Höganäs, Sweden). Total ash content was determined gravimetrically following the methods described by ISO 2171:2010 [26], with incineration at 550 ± 15 °C, 3–5 h, in a Nabertherm calcination furnace (Nabertherm GmbH, Lilienthal, Germany). The results are expressed on a dry matter basis.

The calcium (Ca) content was assessed by a complexometric method, using ethylenediaminetetraacetic acid (EDTA) as a titration agent in the presence of murexide. The analysis of phosphorus (P) content was performed using a spectrophotometric method, employing molybdovanadate and a spectrophotometer Jasco V530 UV/VIS (Japan Servo Co., Ltd., Tokyo, Japan). The copper (Cu), iron (Fe), manganese (Mn), and zinc (Zn) content was determined by flame atomic absorption spectrometry (FAAS, Thermo Electron SOLAAR M6 Dual Zeeman Comfort, Cambridge, UK) after microwave digestion (Berghof, Eningen, Germany), using a digestion and calibration procedure previously validated by Untea et al. [27]. The plant material was analyzed in aliquots of approximately 0.4 g (0.4 ± 2 × 10^−4^ g per sample). Each weighed sample was quantitatively transferred into a 60 mL Teflon DAP-60K digestion vessel together with 7 mL of a 65% HNO_3_: 30% H_2_O_2_ mixture (5:2, *v*/*v*). Mineralization was carried out in a microwave digestion system (BERGHOF, Speedwave MWS-2 Comfort (Eningen, Germany) operating at a maximum power of 1000 W, following a three-stage temperature program: 10 min at 145 °C (80% power), followed by 10 min at 160 °C (80% power), completed with 20 min at 190 °C (80% power). After digestion, the resulting solutions were filtered through filter paper and quantitatively transferred into 50 mL volumetric flasks, which were then made up to volume with deionized water. A reagent blank was processed in parallel, following an identical digestion procedure. An internal validation protocol was developed for mineral determination from plant material using atomic absorption spectrometry (Thermo Electron—SOLAAR M6 Dual Zeeman Comfort (Cambridge, UK). Linearity was established in the range of 0.4–4 ppm for Cu, 0.2–2 ppm for Mn, 0.5–5 ppm for Fe, and 0.1–1 ppm for Zn. The method’s selectivity, accuracy, precision, and recovery were evaluated and validated by applying it on certified reference material bovine liver (CRM—BCR 185R) samples with a complex matrix and comparing the results with the reference values.

### 2.3. Fatty Acid Composition Analyses

The fatty acid composition of hemp seeds was determined by gas chromatography (GC) after conversion of fatty acids to methyl esters (FAME). The extracted fat was mixed with 50 mL of acidified methanol and boiled under reflux in a water bath (FALC WB-U6, FALC Instruments, Treviglio, Italy) for 25–30 min. After cooling, the mixture was combined with distilled water and hexane, stirred, and transferred to a separation funnel. The organic layer was rinsed with distilled water, dried with anhydrous Na_2_SO_4_, and concentrated in a rotary evaporator [28]. Analyses were performed on a PerkinElmer GC-2400 (Waltham, MA, USA). Flame ionization detection was used, with the detector maintained at 300 °C; hydrogen and air flow rates to the detector were set at 35 and 400 mL/min, respectively, and nitrogen served as the make-up gas at 25 mL/min. Separation of the FAME was achieved on an Elite-2560 fused-silica capillary column (PerkinElmer, Waltham, MA, USA; 100 m length, 0.25 mm internal diameter, 0.20 µm film thickness). A stepwise oven temperature program was applied, starting at 60 °C and reaching 240 °C, with heating rates of 15, 6, and 8 °C/min and corresponding hold times of 1, 12, and 8 min. A 1 µL sample volume was introduced via split injection, with the injector port held at 280 °C and hydrogen supplied as carrier gas under constant pressure (35 psi). Peaks corresponding to individual FAMEs were assigned based on retention-time matching against a Supelco 37-component FAME reference standard (Merck, Darmstadt, Germany), without individual response-factor correction. Percentages were calculated from normalized FID peak areas, a standard simplification for routine FA profiling. The amount of each FA was expressed as g/100 g FAME, after triplicate analyses and readings.

### 2.4. Hydrosoluble Antioxidant Compounds Extraction and Analyses

Hydrosoluble antioxidants were extracted from hemp seeds by mixing 1 g of dried, ground sample with 20 mL of 80% methanol. The mixture was continuously agitated in a rotary shaker for 24 h at room temperature in the dark and subsequently centrifuged at 1500× *g* for 10 min (Sigma 3–16 KL, Sigma Laborzentrifugen GmbH, Osterode am Harz, Germany). The resulting supernatant was collected and used for subsequent analyses [29].

Total phenolic content (TPC) in the hemp seed extracts was determined spectrophotometrically using a Folin–Ciocalteu assay adapted with minor modifications by Untea et al. [29]. For each measurement, 0.5 mL of extract (at appropriate dilutions) was combined with 0.5 mL Folin–Ciocalteu reagent and 7 mL distilled water, and the mixture was thoroughly homogenized. Following 3 min incubation at room temperature, 2 mL of 20% sodium carbonate solution was added. The reaction was allowed to proceed in the dark for 60 min, and absorbance was then recorded at 732 nm using a Varioskan Lux microplate reader (Thermo Fisher Scientific, Waltham, MA, USA) relative to a reagent blank (extract-free solution). Total phenolic content was quantified against a gallic acid calibration curve (R^2^ = 0.9993) and expressed as milligrams of gallic acid equivalents per gram of sample (mg GAE/g dw).

The free radical-scavenging capacity of the extracts was assessed via a DPPH assay, as described by Untea et al. [29]. Briefly, 400 μL of supernatant was combined with 1.6 mL distilled water and 2 mL of a 0.2 mM methanolic DPPH solution. After vortexing, the mixture was left to stand in the dark at ambient temperature for 30 min. A control tube, prepared with 2 mL methanol in place of the sample, was analysed simultaneously. Absorbance readings were taken at 517 nm on a Varioskan Lux microplate reader (Thermo Fisher Scientific, Waltham, MA, USA). The calibration curve was constructed using various concentrations (0.5–25 mg/L) of Trolox (6-hydroxy-2,5,7,8-tetramethylchroman-2-carboxylic acid) and the antioxidant activity of the samples was expressed as millimoles of Trolox equivalent (mmol TE/kg dw).

The 2,2′-azino-bis-3-ethylbenzthiazolin-6-sulfonic acid (ABTS) antioxidant activity of samples was determined as described elsewhere [30]. The absorbance of samples against a blank was read at 734 nm using a Varioskan Lux microplate reader (Thermo Fischer Scientific Waltham, MA, USA). The calibration curve was prepared by plotting the absorbance at 734 nm versus different concentrations of Trolox (6-hydroxy-2,5,7,8-tetramethylchroman-2-carboxylic acid). The results were quantified as millimoles of Trolox equivalent (mmol TE/kg dw).

Total antioxidant capacity was determined using the phosphomolybdenum method [29]. An aliquot of 0.2 mL of extract was mixed with 4 mL of reagent solution containing 0.6 M sulfuric acid, 28 mM sodium phosphate, and 4 mM ammonium molybdate. The absorbance was recorded at 695 nm with a Varioskan Lux microplate reader (Thermo Fischer Scientific, Waltham, MA, USA). The reagent blank contained 0.2 mL of 80% methanol. Quantification was performed using an ascorbic acid calibration curve. Results were expressed as mmol equivalents ascorbic acid per kilogram of sample dry weight (mmol AAE/kg dw).

For determination of the chelating effect on ferrous ions, the Varioskan Lux microplate reader (Thermo Fisher Scientific, Waltham, MA, USA) was used to measure the absorbance of the purple color of the complex formed when an extract competed with ferrozine for ferrous ions against the blank at 562 nm. The calibration curve (R^2^ = 0.9971) was prepared by plotting the absorbance at 562 nm versus different concentrations of ethylenediaminetetraacetic acid (EDTA). The chelating effect on ferrous ions was quantified as mg EDTA equivalents per gram of the sample (mg EDTA/g dw).

### 2.5. Liposoluble Antioxidant Compounds Extraction and Analyses

The extraction of liposoluble antioxidants from hemp seeds followed the procedure previously described by Varzaru et al. [31]. Prior to extraction, a saponification step was necessary, involving the hydrolysis of samples with an ethanolic potassium hydroxide solution in a water bath for 30 min at 80 °C, followed by petroleum ether extraction.

The carotenoid content was determined using a high-performance liquid chromatography (HPLC) method with a PerkinElmer Series 200 system (Shelton, CT, USA) equipped with a UV detector set at 450 nm. The carotenoids were identified and quantified using individual analytical standards: lutein (95%, Sigma-Aldrich, Burlington, MA, USA), astaxanthin (97%, Sigma-Aldrich), and canthaxanthin (95%, Sigma-Aldrich). The mobile phase consisted of acetone, methanol, and water (75:15:10, *v*/*v*/*v*), and separation was performed on a Nucleodur C18 reversed-phase column (5 µm, 250 × 4.6 mm internal diameter; Macherey-Nagel, Düren, Germany), according to a method described previously [31]. The chromatographic analysis was performed at a flow rate of 0.5 mL/min, using an injection volume of 25 µL and a total run time of 15 min. The column was not thermostatically controlled, and the analyses were conducted at ambient laboratory temperature. Linearity was evaluated over concentration ranges of 10–50 µg/mL for lutein, 2.5–7.5 µg/mL for astaxanthin, and 2.5–7.5 µg/mL for canthaxanthin. Quantification was performed using external calibration curves prepared from the corresponding individual analytical standards. Lutein was identified by retention-time matching with the corresponding analytical standard and UV detection at 450 nm. The assignments of astaxanthin and canthaxanthin were based on retention-time matching with their corresponding analytical standards and UV detection at 450 nm, without LC-MS/MS confirmation; therefore, these two compounds were considered tentatively assigned. The results were expressed as milligrams per kilogram of sample (mg/kg).

Individual tocopherols were determined using a Vanquish Core HPLC system (Thermo Fisher Scientific, Waltham, MA, USA) equipped with a PDA–UV detector set at 292 nm. Separation was performed on an Accucore C18 column (4 µm particle size, 150 × 4.6 mm internal diameter; Thermo Fisher Scientific, Waltham, MA, USA), using a mobile phase consisting of methanol and water (96:4, *v*/*v*). The chromatographic analysis was performed at a mobile-phase flow rate of 0.5 mL/min, using an injection volume of 40 µL, a column temperature of 25 °C, and a total run time of 11 min. Linearity was established over the concentration range of 0.5–10 µg/mL for the analyzed tocopherols. The concentrations of the individual tocopherols were expressed as milligrams per kilogram of sample (mg/kg), and total tocopherol content was calculated as the sum of the quantified α-, γ-, and δ-tocopherol concentrations.

### 2.6. Statistical Analyses

Statistical analysis was performed by one-way ANOVA using the XLStat statistical software package (version 2021.3), followed by Tukey’s post hoc test. Each cultivar was represented by three independently sampled field plots (biological replicates, n = 3), and each plot-level sample was analyzed in triplicate (technical replicates), as described in Section 2.1. For each analytical variable, the three technical determinations obtained from each plot-level sample were averaged to produce a single value per biological replicate. One-way ANOVA was performed using the three independent plot-level means for each cultivar (n = 3), and technical replicates were not entered as independent observations. Principal component analysis (PCA) was performed to explore the relationships among the analyzed variables using the corresponding function in XLStat (version 2021.3). Graphs were created using GraphPad Prism, version 9.03 (GraphPad Software, San Diego, CA, USA).

## 3. Results

### 3.1. Nutritional Composition of the Romanian Hemp Seed Varieties

The nutritional composition of the six monoecious Romanian hemp seed varieties analyzed showed significant differences among the nutritional parameters. DM was constant among the hemp seed varieties. The CP content was significantly higher (*p* < 0.001) in Dacia Secuieni, Secuieni Jubileu, Ratza, and Olivia, compared with Succesiv and Zenit. The EE content ranged significantly (*p* < 0.001) between Succesiv and Secuieni Jubileu. The highest difference among varieties was observed for CF content. Ratza and Zenit exceeded 37% CF while Dacia Secuieni and Secuieni Jubileu were below 32%. The ash content was significant (*p* < 0.001), especially between Olivia, Ratza, and Zenit compared with Dacia Secuieni, followed by Secuieni Jubileu and Succesiv hemp seed (Table 1).

### 3.2. Mineral Composition of the Romanian Hemp Seed Varieties

Within the mineral composition (Table 2), the Ca content showed no significant differences (*p* = 0.082), while P content was significantly different only between Dacia Secuieni and Ratza (*p* = 0.049). All trace minerals evaluated showed significant differences (Cu, Fe, Mn: *p* < 0.0001; Zn: *p* = 0.022). The lowest Cu concentrations were determined in Olivia and Succesiv, while the highest was noted in Ratza hemp seeds. Succesiv showed the lowest Fe content, while Zenit and Secuieni Jubileu exhibited significantly higher concentrations (*p* < 0.0001). Mn showed the greatest variability among hemp seed varieties, ranging from 91.59 mg/kg in Olivia to 138.91 mg/kg in Dacia Secuieni. Zn showed an opposite trend in its content deposition, with Olivia exhibiting the highest content (*p* = 0.022) while Succesiv and Secuieni Jubileu had the lowest.

### 3.3. Fatty Acids Composition of the Romanian Hemp Seed Varieties

The FA composition of the six monoecious Romanian hemp seed varieties is presented in Table 3. The predominant lipid fraction in all seeds was represented by PUFAs, ranging from 72.74% of total identified FAs in Ratza to 78.10% in Succesiv (*p* < 0.001). Dacia Secuieni exhibited the highest ALA concentration (19.76 g/100 g FAME), while Secuieni Jubileu, Zenit, Succesiv, and Olivia averaged 16.22% and Ratza had the lowest at only 15.20%. Succesiv and Olivia showed the highest LA concentrations (56.11% and 56.16%) compared with the other four cultivars. Oleic acid (C18:1n-9), the major MUFA, varied significantly (*p* < 0.001) from 11.84% in Succesiv to 16.47% in Ratza. Among bioactive FAs, γ-linolenic acid (GLA; C18:3n-6) was significantly higher in Zenit (4.307%), followed by Succesiv (4.116%), compared with Dacia Secuieni (2.055%) and Ratza (1.930%). Palmitic (C16:0) and stearic (C18:0) FAs were the predominant SFAs. Ratza (6.977%) and Olivia (6.852%) contained higher palmitic acid. Secuieni Jubileu (2.823%) and Ratza (2.741%) contained higher stearic acid. The distribution of the two major PUFAs (LA and ALA) differed significantly among varieties. Dacia Secuieni showed the highest omega-3 content (20.35%), despite not having the highest PUFA concentration. This resulted in the lowest omega-6/omega-3 ratio (2.78; *p* < 0.0001). In contrast, Ratza had the lowest omega-3 concentration (15.68%), resulting in the highest omega-6/omega-3 ratio (3.64). Succesiv, Zenit, and Olivia had close omega-6/omega-3 ranging values.

### 3.4. Liposoluble Bioactive Compounds of the Romanian Hemp Seed Varieties

Significant differences were observed for all liposoluble antioxidant compounds evaluated (Table 4). The highest total tocopherol content was determined in Dacia Secuieni (227.44 mg/kg), followed by Secuieni Jubileu (222.22 mg/kg), while Olivia had the lowest content (171.37 mg/kg). γ-tocopherol represented the predominant tocopherol isoform in all varieties. Significantly lower (*p* < 0.001) content was determined in Olivia compared with the highest content in Dacia Secuieni. The α-tocopherol isoform was significantly higher in Secuieni Jubileu, followed by Ratza and Dacia Secuieni, compared with only 21.94 mg/kg in Succesiv. Among carotenoids, lutein was detected in significantly higher (*p* < 0.0001) concentrations in all seeds compared with the compounds tentatively assigned as astaxanthin and canthaxanthin. Lutein ranged from 47.73 mg/kg in Secuieni Jubileu to 79.46 mg/kg in Succesiv. The peak tentatively assigned as astaxanthin showed the highest numerical value in Zenit (1.32 mg/kg), although this value did not differ significantly from those recorded for Dacia Secuieni (1.30 mg/kg) and Ratza (1.29 mg/kg). The lowest value was observed in Secuieni Jubileu (0.91 mg/kg). For the peak tentatively assigned as canthaxanthin, Ratza showed the highest numerical value, without significant differences from Succesiv and Dacia Secuieni, whereas Olivia exhibited the lowest value.

### 3.5. Hydrosoluble Bioactive Compounds of the Romanian Hemp Seed Varieties

The measurements of hydrosoluble antioxidant compounds are presented in Table 5. Zenit showed the highest TPC (1.50 mg GAE/g), followed by Succesiv (1.39 mg GAE/g dw) and Dacia Secuieni (1.274 mg GAE/g dw), whereas Secuieni Jubileu and Ratza exhibited the lowest concentrations. In contrast, the highest DPPH activity was observed in Succesiv (12.89 mmol TE/kg dw), Dacia Secuieni (12.80 mmol TE/kg dw), and Secuieni Jubileu (12.54 mmol TE/kg dw). Although Olivia showed the lowest DPPH antioxidant activity (7.91 mmol TE/kg dw) and metal chelating activity (2.21 mg EDTA/g), it was noted to have the highest (*p* < 0.001) total antioxidant capacity (49.70 mmol AAE/kg dw) determined by the phosphomolybdenum method. Regarding ABTS radical-scavenging activity, Zenit recorded significantly higher (14.97 mmol TE/kg dw) values than the other five cultivars (*p* < 0.001).

### 3.6. Principal Component Analysis (PCA)

To explore multivariate relationships among cultivars while avoiding compositionally redundant variables, PCA was performed on 14 independent variables, selected from nutritional composition (CP, EE, and CF), minerals (Fe, Mn, Zn), liposoluble bioactives (total tocopherol and lutein), hydrosoluble antioxidant activity (TPC, DPPH, ABTS), and individual fatty acids (LA, GLA, ALA), using the 18 plot-level biological replicates (six cultivars × three independently sampled field plots), after standardization to unit variance (Table 6). The first two principal components (F1 and F2) accounted for 36.0% and 22.4% of total variance, respectively (58.4% cumulative; Table 6), while F3 retained 18.1% (Figure 1). F1 was defined by strong positive loadings for LA (0.885), CF (0.696), lutein (0.624) and GLA (0.568), opposed to strong negative loadings for Mn (−0.806), CP (−0.752), tocopherol (−0.730), EE (−0.726), and ALA (−0.607), separating Zenit, Succesiv, and Olivia (positive F1) from Dacia Secuieni, Secuieni Jubileu, and Ratza (negative F1). F2 was characterized by strong positive loadings for DPPH (0.869), GLA (0.592), tocopherol (0.548) and Mn (0.526), and a strong negative loading for Zn (−0.773), separating Succesiv (highest F2) from Olivia and Ratza (lowest F2). F3 was dominated by Fe (0.811) and ABTS (0.807), clearly separating Zenit (F3 = 3.13) from all the other cultivars. Plot-level replicates clustered tightly within each cultivar relative to the distances separating cultivars (Figure 1), indicating that the observed multivariate separation reflects consistent cultivar-level differences. Cultivar-mean scores were as follows: Dacia Secuieni (F1 = −3.44, F2 = 0.50, F3 = −0.90), Secuieni Jubileu (−2.26, 0.62, 0.79), Ratza (−0.38, −1.71, −0.44), Zenit (1.46, 0.46, 3.13), Succesiv (2.56, 2.76, −1.71), and Olivia (2.06, −2.63, −0.87).

## 4. Discussion

### 4.1. Nutritional Composition of the Monoecious Romanian Hemp Seed Varieties

The CP content showed significant differences among the Romanian hemp seed varieties, but the values were comparable with those reported for European and Canadian industrial hemp seeds [12,17,32]. From a food formulation perspective, differences in CP content are very important when hemp seeds are incorporated into protein-enriched products. The higher CP observed in Ratza and Dacia Secuieni may increase the nutritional density of food products while reducing the amount of raw material required to achieve the target CP content. Previous studies have reported that hemp seed proteins are rich in edestin and albumin, which increase digestibility and favor techno-functional properties (water absorption, emulsification, and foaming capacity) when incorporated in food products [33]. Furthermore, the higher fat content, particularly in Secuieni Jubileu and Dacia Secuieni, may be better suited for cold-pressed oil production and premium functional foods enriched with essential FAs. In contrast, the Succesiv cultivar, which contained the lowest fat content, represents a candidate source for protein-rich flour production following oil extraction. This distinction is particularly relevant considering the rapid expansion of the plant-based protein market, where partially defatted hemp seed flour is increasingly utilized as a sustainable ingredient in bakery and snack products [7,9]. Dietary fiber is recognized more broadly as a desirable nutritional attribute, associated in the literature with improved gastrointestinal function, glycemic regulation, cholesterol metabolism, and satiety [31]. Ratza and Zenit hemp seeds, which showed the highest crude fiber content, may be of particular interest for further development of high-fiber foods. Ingredients rich in fibers have shown technological advantages (water retention, texture, shelf-life) in bakery applications, according to other studies [34]. The elevated CF content observed in these cultivar seeds represents not only a nutritional advantage but also a functional characteristic that may enhance product quality. In terms of ash content, the Dacia Secuieni cultivar included a greater proportion of essential minerals required in the human diet, which may enhance the nutritional value of food formulations incorporating this cultivar. Similar variation in mineral accumulation among different hemp seed cultivars was previously reported by Irakli et al. [12], who demonstrated significant differences in macro- and microelement concentrations in hemp seed varieties cultivated under comparable environmental conditions. Such variability provides opportunities for targeted cultivar selection according to the intended food application, supporting the growing concept of nutrition-oriented breeding and the development of hemp seed-based ingredients tailored to specific consumer and industrial needs.

### 4.2. Mineral Composition of the Monoecious Romanian Hemp Seed Varieties

In terms of mineral content, the results highlighted that the evaluated Romanian monoecious hemp seed cultivars represent valuable sources of dietary minerals, although cultivar-dependent variability was observed. This is in line with previous reports on Canadian, Greek, Italian, and Nordic–Baltic region hemp seeds, suggesting that mineral accumulation is strongly influenced by genetic background as well as environmental factors [12,17]. The Ca content showed little variability among cultivars (0.215% to 0.247%), however, the content was higher than the values obtained in the Nordic–Baltic region (0.10–0.19%) [35]. The stable Ca content observed among Romanian hemp seed cultivars suggests that Ca is less influenced by cultivar compared with trace mineral accumulation. The highest P concentration was found in Dacia Secuieni (1.32%) and the lowest in Ratza (0.54%). The P content determined in Romanian cultivars was double than those reported by Barčauskaitė et al. [36] in five hemp seed cultivars (0.41% to 0.51%), and comparable with those reported by Irakli et al. [12] (0.70%) and House et al. [32] (1.20%). Considering that the recommended P intake for adults according to National Institutes of Health is 700 mg/day, the European Union (EFSA) recommends 550 mg/day and Nordic countries 600 mg/day [37,38], a 30 g serving of Dacia Secuieni would provide approximately 397 mg of P (1.322 g/100 g × 30 g), corresponding to approximately 72% of the EFSA adequate intake (550 mg/day) or 57% of the U.S. reference intake (700 mg/day). Among the microelements, Fe and Mn were the most abundant, followed by Zn and Cu. From these elements, Fe content requires particular attention due to its public health relevance. Low Fe intake remains the most prevalent micronutrient deficiency worldwide and continues to affect more than one billion individuals [39,40]. The Fe concentrations observed in the present study ranged between 70.91 mg/kg (Succesiv) to 105.21 mg/kg (Zenit), all six being within or above the ranges reported for other commercial seed cultivars [12,39]. In hemp seeds from the Nordic–Baltic region, Barčauskaitė et al. [36] also reported that Fe was the most abundant microelement, however, the content was higher (181 to 334 mg/kg). Similarly, Arango et al. [41] reported that Fe content in 29 monoecious and dioecious hemp seed cultivars from eight different EU countries ranged between 58.5 mg/kg and 323.7 mg/kg. From a practical perspective, a 30 g serving of Zenit seeds would provide approximately 3.2 mg of Fe, representing about 18% to 35% of the RDI depending on age and sex [39]. Consequently, hemp seeds from Zenit and Secuieni Jubileu cultivars might be particularly attractive for vegetarian, vegan, and flexitarian products designed to potentially enhance dietary Fe intake, based on their compositional Fe content. However, the actual nutritional contribution depends on mineral bioavailability, which is influenced by food processing conditions and by phytate and other antinutrients known to occur in hemp seed. Further, the Mn levels ranged from 92 mg/kg to 139 mg/kg, exceeding values reported for other cereals and legumes [42]. This is of importance since Mn functions as a cofactor for numerous enzymes involved in carbohydrate metabolism, bone formation, mitochondrial function, and antioxidant defense through manganese superoxide dismutase [40,43]. According to the EFSA Panel on Dietetic Products, Nutrition and Allergies [44], the adequate intake for adults is 3 mg/day. A 30 g serving of Dacia Secuieni would provide approximately 4.2 mg of Mn, covering or exceeding the RDI. In contrast, Alonso-Esteban et al. [45] reported a much lower average Mn concentration of approximately 5.18 mg/100 g in eight hulled hemp seed varieties cultivated in Spain. The Romanian monoecious cultivars therefore show substantially higher manganese content than this comparator. The content of Cu was higher in Ratza, Secuieni Jubileu, and Zenit varieties, which contained over 15 mg/kg. This corresponds to approximately 0.50 mg of Cu per 30 g serving, nearly half of the 0.90 mg/day adequate intake for adults. Similarly, Alonso-Esteban et al. [45] reported that in eight hemp seed Spanish cultivars, the Cu content was lower than other elements, as in our case. The authors reported an average of 2.30 mg/100 g in whole hemp seeds, while lower content was noted in dehulled hemp seeds (1.48 mg/100 g). In human diets, Cu plays critical roles in energy metabolism, connective tissue synthesis, neurological function, and antioxidant defense through its involvement in Cu/Zn-SOD activity [46]. Regarding Zn content, the most notable amount was determined in Olivia (90.35 mg/kg), Ratza (79.12 mg/kg), and Dacia Secuieni (78.45 mg/kg) hemp seed cultivars. Zn is an essential trace element involved in over 300 enzymatic reactions, with essential roles in immune function, protein synthesis, DNA repair, and cellular growth. These three cultivars can provide a significant amount of Zn content in terms of the RDI, especially for consumers following plant-based diets. The substantial concentrations of Fe, Mn, Cu, and Zn strengthen the potential of hemp seeds as functional food ingredients that may contribute to current consumer demands for nutrient-dense, plant-based, and minimally processed foods.

### 4.3. Fatty Acids Composition of the Monoecious Romanian Hemp Seed Varieties

The lipid composition in all the tested Romanian hemp seeds varieties revealed an FA profile dominated by PUFAs. This confirms previous reports which showed that hemp seeds are among the richest source of essential FA available for human consumption [47,48,49,50]. LA was the dominant FA, accounting for up to 57% of total FAs. These values are consistent with, and in several cases higher than, those reported for other hemp seed varieties, where LA ranged between 50 and 56% [45,50,51]. LA is an essential omega-6 FA that cannot be synthesized by humans and serves as a precursor for numerous signaling molecules involved in immune function, skin integrity, and cellular metabolism, and which adds nutritional contribution when consumed. Among the omega-3 FAs, the most abundant was ALA, which reached 20% of total FAs in Dacia Secuieni. This content exceeds the values reported by Rosso et al. [50] in Italian hemp seed cultivars and is comparable with Polish varieties [52]. ALA is the primary plant-derived omega-3 FA, serving as a precursor for the endogenous synthesis of eicosapentaenoic acid (EPA) and docosahexaenoic acid (DHA). Although conversion efficiency in humans remains limited, increasing dietary ALA intake has consistently been associated with improved health outcomes when regularly consumed [17,53]. The elevated ALA concentrations determined in Dacia Secuieni represent a particularly valuable nutritional characteristic from both consumer and food industry perspectives. In addition to essential FAs, hemp seeds are distinguished by the presence of GLA, a relatively rare bioactive FA found in a limited number of edible plant oils. The concentrations observed in the present study, particularly in Zenit (4.307%) and Succesiv (4.116%), are in line with others [52,54]. The bioactivity of this FA is given by the fact that it serves as a precursor of dihomo-γ-linolenic acid, which participates in the synthesis of anti-inflammatory eicosanoids. Although present at lower concentrations, stearidonic acid (SDA) was determined in all these Romanian hemp seed varieties, with Succesiv showing the numerically highest level, although the difference was not statistically significant (*p* = 0.081). This FA is increasingly recognized as one of the most valuable FAs in hemp oil because it bypasses the rate-limiting Δ6-desaturase step required for conversion of ALA into longer-chain omega-3 FAs [55]. Consequently, SDA exhibits substantially higher conversion efficiency to EPA than ALA alone. Since only a few edible plant species naturally contain SDA, its presence further differentiates hemp oil seed from other vegetable oils commonly used in human nutrition. Furthermore, total SFA content was lower in all hemp seed varieties, which highlights another favorable nutritional attribute. Palmitic and stearic acids together accounted for less than 10% of total FAs, values generally lower than those reported for other consumed edible oil seeds [17]. In terms of public health perspective, replacing dietary SFAs with PUFAs has consistently been associated with reductions in cardiovascular disease risk and improved plasma lipid profiles [56]. Regarding the PUFA content, in addition to ALA, C22:5n-3 was also detected at trace levels (0.045–0.077% of total FAs); however, its detection is regarded as tentative due to possible co-elution with other trace/odd-chain constituents. Based on the significant concentrations of LA and ALA, all investigated hemp seed varieties exhibited favorable omega-6/omega-3 ratios close to the range of 2.7:1–3.6:1, which falls within the range often discussed as favorable in the literature. Modern Western diets frequently exceed ratios of 15–20:1, a pattern associated with chronic inflammation and increased risk of metabolic disorders [57]. Therefore, the lipid composition observed in the investigated hemp seed varieties aligns well with current nutritional recommendations promoting increased consumption of unsaturated FAs. Consequently, incorporation of hemp seeds into daily diets may contribute to restoring a more balanced intake of essential FAs. From an industrial perspective, cultivar selection may enable targeted applications according to the desired nutritional profile. Dacia Secuieni might be particularly suitable for the production of omega-3-enriched ingredients due to its superior ALA concentration. In contrast, Zenit may be advantageous for the development of functional foods emphasizing GLA intake, while Ratza could be preferred when higher oleic acid concentrations are desirable due to their potential influence on oxidative stability.

### 4.4. Liposoluble and Hydrosoluble Compounds Monoecious Romanian Hemp Seed Varieties

The liposoluble antioxidant fraction of hemp seeds contributes to protecting the abundant PUFA content against oxidative degradation and adds further nutritional and functional value to hemp seed-derived food products. In agreement with previous reports [12,13,57], γ-tocopherol was confirmed as the predominant tocopherol isoform in all six Romanian hemp seed varieties (130.35–175.78 mg/kg), followed by α-tocopherol (21.94–31.14 mg/kg) and δ-tocopherol (14.82–23.37 mg/kg). These γ-tocopherol concentrations were significantly higher than those reported by Irakli et al. [12] for seven Greek hemp genotypes (46–113 mg/kg), and they are comparable with the mean value reported by Kriese et al. [58] across 51 hemp genotypes of diverse geographical origin (approximately 217 mg/kg). This indicates that in addition to the cultivar, growing conditions and extraction/analytical methods influences the wide range of tocopherol values reported for hemp seeds. Regarding the FA profiles, Dacia Secuieni contained the highest total tocopherol content with a favorable FA composition, which may potentially confer greater oxidative stability to oil and food products derived from this cultivar, though this was not directly measured in this study; this remains an attribute of interest given the high PUFA content of hemp seed oil. Among carotenoids, lutein was the predominant pigment in all varieties (47.73–79.46 mg/kg), being two- to five-fold higher than the values reported by Irakli et al. [12] for Greek hemp genotypes (15 to 34 mg/kg) and within the upper range summarized in recent reviews of hemp seed composition. Lutein is a xanthophyll carotenoid with recognized roles in ocular health, protecting macular cells against light-induced oxidative stress, which further supports the potential of designing hemp-derived ingredients for functional food development [59]. The astaxanthin and canthaxanthin concentrations determined in the present study, although significantly different among cultivars, could not be directly benchmarked against the existing hemp seed literature, since these ketocarotenoids, more typically associated with microalgae, crustaceans, and certain yeasts, have rarely been reported or quantified in *Cannabis sativa* seeds. A previous study on hemp seed-derived matrices also investigated the astaxanthin and canthaxanthin content [60]. However, neither compound was detected in the analyzed samples; it is suggested that this is related to possible carotenoid degradation during the oil extraction process. Given the unusual detection of these tentatively identified ketocarotenoids in the present study, further LC-MS-based investigations might provide additional insights into their occurrence in this matrix.

Alongside the liposoluble bioactive compounds, the hydrosoluble antioxidant fraction represents another important quality attribute of hemp seeds for food applications. The TPC values obtained for the six Romanian hemp seed varieties (0.74 to 1.50 mg GAE/g) were generally lower than those reported by Vonapartis et al. [17] for ten Canadian hemp seed samples (1.37 to 5.16 mg GAE/g) and by Irakli et al. [12] for seven types of Greek hemp seed, while they were comparable with the range reported by Siano et al. [13] for the Fedora cultivar (0.77 mg GAE/g dw) and by Rosso et al. [50] for three varieties of Italian hemp seeds. More recently, Floareș Oarga et al. [18] reported similar TPC values (0.73 to 1.52 mg GAE/g) obtained through combined maceration and ultrasound-assisted extraction, a technique the authors showed to increase TPC recovery by up to 55% compared with conventional solvent extraction. This indicates that, in addition to the cultivar, the extraction protocol strongly influences the measured phenolic content and should be considered when comparing hydrosoluble antioxidant values across studies. The lack of a consistent relationship between TPC and the DPPH/ABTS radical-scavenging activities observed in the present study is in agreement with previous reports [12,50] where it was similarly reported that the antioxidant capacity of hemp seed extracts was not driven only by the total phenolic pool but also reflected the individual reducing power of specific phenolic acids (e.g., N-trans-caffeoyltyramine), flavonoids, and their interaction with other reducing compounds co-extracted from the seed matrix. From a food-application perspective, Zenit and Succesiv seeds, which combined the highest TPC with high DPPH and ABTS activity, appear to be the most suitable cultivars for the development of antioxidant-rich, minimally processed hemp-based ingredients, while the remaining cultivars, despite lower phenolic contents, may still contribute meaningfully to the overall antioxidant capacity of hemp-based food formulations through their liposoluble tocopherol and carotenoid fractions, as described above.

### 4.5. Principal Component Description

The PCA performed provided an exploratory representation of both between-cultivar differentiation and within-cultivar dispersion. The first three principal components accounted for a substantial proportion of the total variance, with F1 primarily describing a compositional gradient between cultivars characterized by higher LA, CF, lutein, and GLA and those associated more strongly with CP, EE, Mn, total tocopherols, and ALA. Along this axis, Zenit, Succesiv, and Olivia were positioned toward the positive side, whereas Dacia Secuieni and Secuieni Jubileu exhibited negative scores. Ratza occupied a comparatively intermediate position, reflecting its mixed profile, particularly its high CF and lutein concentrations but relatively low GLA and ALA levels. Regarding F2, Succesiv showed the highest cultivar centroid score (2.76), consistent with its combination of high DPPH radical-scavenging activity, GLA, and lutein concentrations. DPPH activity exhibited the strongest positive loading on this component, while GLA, total tocopherols, Mn, ABTS activity, and lutein also contributed positively to the observed pattern. This position is consistent with the antioxidant and compositional characteristics described in Section 3.4 and Section 3.5. Conversely, Olivia and Ratza displayed the lowest F2 scores. Their negative positions were associated primarily with comparatively low DPPH activity and, for Ratza, lower ABTS activity and GLA concentration. In Olivia, the negative F2 position was also associated with its high Zn concentration, as Zn loaded negatively on this component. F3 provided an additional distinction separating Zenit from the other cultivars, with a cultivar centroid score of 3.13. This separation was associated predominantly with the positive loadings of Fe and ABTS radical-scavenging activity, together with contributions from EE and GLA. This multivariate pattern agrees with the univariate findings in which Zenit exhibited the highest Fe concentration and the strongest ABTS radical-scavenging activity (Section 3.2 and Section 3.5). Nevertheless, because PCA is an exploratory method, these results are interpreted as descriptive multivariate support rather than as confirmatory evidence. Overall, no cultivar occupied a uniformly favorable position across all three components. This finding sustains the notion that cultivar-specific selection, rather than identification of a single overall best cultivar, represents the most appropriate strategy for targeted food applications.

## 5. Conclusions

This study demonstrates that the six Romanian monoecious hemp seed cultivars evaluated possess distinct nutritional and bioactive profiles, supporting cultivar-specific selection for targeted food applications. Dacia Secuieni was distinguished by its high protein and fat contents, phosphorus, manganese, ALA, omega-3, and total tocopherol levels, and the lowest omega-6/omega-3 ratio. Secuieni Jubileu contained the highest crude fat and α-tocopherol concentrations and was among the cultivars richest in protein and iron. Ratza also contained the high crude fibre, copper, oleic acid, SFA, and MUFA contents, but had the lowest PUFA and omega-3 levels. Zenit was characterized mostly by high iron, GLA, TPC, and ABTS values. Succesiv was characterized mostly by PUFA, omega-6, lutein, DPPH, and Fe^2+^-chelating values, despite having the lowest protein and fat contents. Olivia was distinguished by the highest zinc, LA, and total antioxidant capacity, but contained the lowest total tocopherol concentration. Overall, no single cultivar was superior in all parameters. The observed differences support cultivar-specific selection but require confirmation in multi-year and multi-location trials.

## Figures and Tables

**Figure 1:**
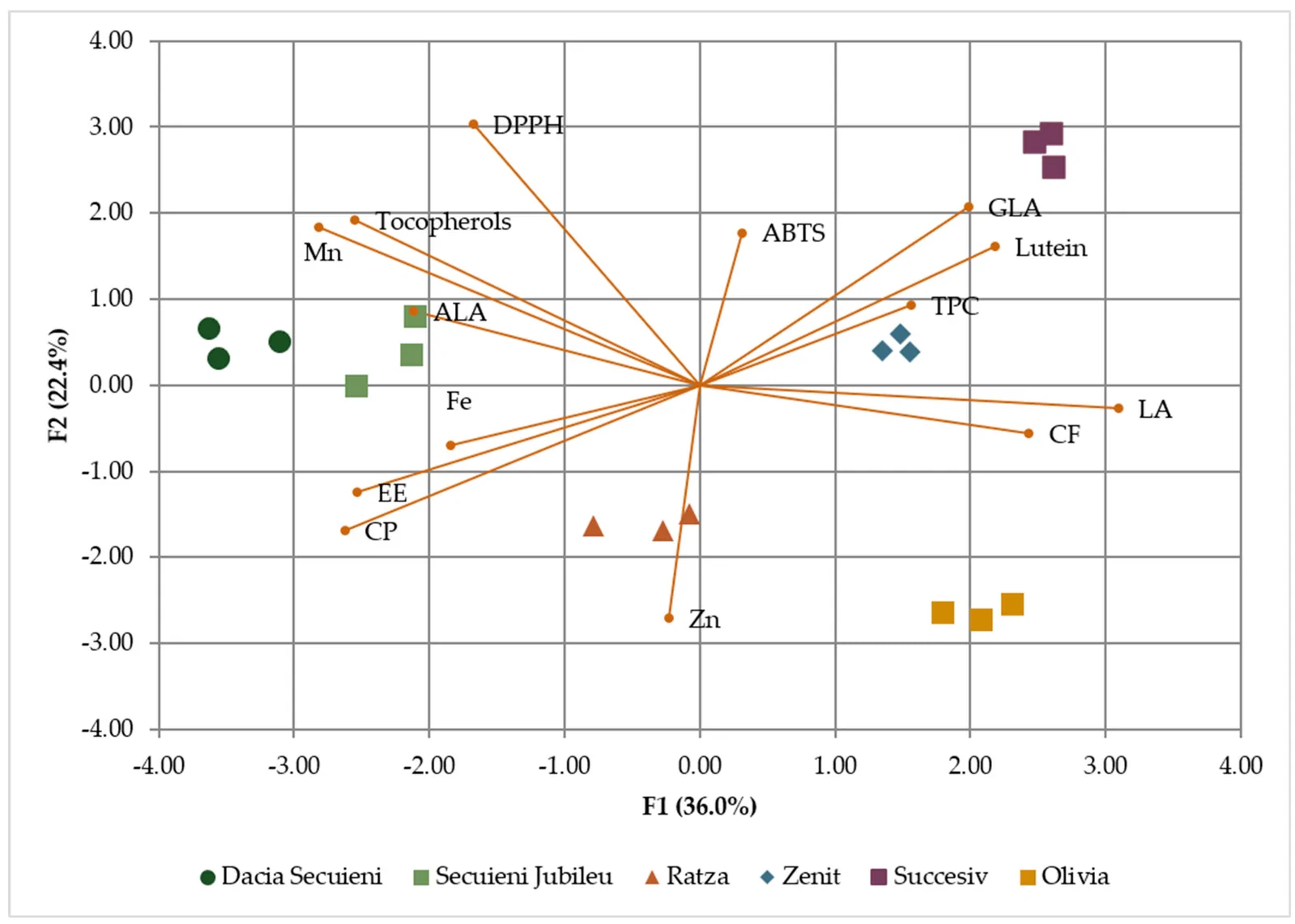
PCA biplot. F1 and F2 together accounted for 58.40% of the total variance in the standardized dataset. Plot-level observations (n = 18; 6 cultivars × 3 field plots). F1—first principal component (36.00% of the total variance); F2—second principal component (22.34%); CP—crude protein; EE—ether extract; CF—crude fiber; Fe—iron; Mn—manganese; Zn—zinc; TPC—total phenolic content; DPPH—2,2-diphenyl-1-picrylhydrazyl radical-scavenging activity; ABTS—2,2′-azino-bis(3-ethylbenzothiazoline-6-sulfonic acid) radical-scavenging activity; LA—linoleic acid (C18:2n-6); GLA—γ-linolenic acid (C18:3n-6); ALA—α-linolenic acid (C18:3n-3).

**Table 1:** Nutritional composition of *Cannabis sativa* seeds.

Parameters	DM	CP	EE	CF	Ash
	%	%	%	%	%
Dacia Secuieni	94.50 ^a^	25.54 ^a^	29.46 ^a^	31.64 ^e^	6.03 ^a^
Secuieni Jubileu	95.85 ^a^	24.89 ^a^	30.96 ^a^	30.99 ^e^	5.34 ^b^
Ratza	94.41 ^a^	25.57 ^a^	29.67 ^a^	38.85 ^a^	4.48 ^c^
Zenit	95.57 ^a^	24.07 ^b^	30.10 ^a^	37.97 ^b^	4.72 ^c^
Succesiv	95.58 ^a^	23.96 ^b^	25.62 ^c^	36.25 ^c^	5.37 ^b^
Olivia	95.84 ^a^	24.79 ^a^	28.07 ^b^	34.78 ^d^	4.29 ^c^
SEM	1.372	0.170	0.226	0.142	0.131
R^2^	0.953	0.866	0.966	0.995	0.895
*p* value	0.093	<0.001	<0.0001	<0.0001	<0.001

Different letters (a, b, c, d, e) within each column indicate significant difference at *p* < 0.05 between seed cultivars; DM—dry matter; CP—crude protein; EE—crude fat; CF—crude fiber; SEM—standard error of the mean; R^2^—coefficient of determination of the ANOVA model; *p*—significance level according to ANOVA test; n = 3 biological replicates per cultivar.

**Table 2:** Mineral composition of *Cannabis sativa* seeds.

Parameters	Ca	P	Cu	Fe	Mn	Zn
	%	%	mg/kg	mg/kg	mg/kg	mg/kg
Dacia Secuieni	0.246 ^a^	1.322 ^a^	13.62 ^d^	91.44 ^c^	138.91 ^a^	78.45 ^ab^
Secuieni Jubileu	0.215 ^a^	1.184 ^ab^	15.96 ^b^	102.44 ^a^	126.50 ^b^	55.36 ^c^
Ratza	0.222 ^a^	0.541 ^b^	16.17 ^a^	97.58 ^b^	113.70 ^d^	79.12 ^ab^
Zenit	0.221 ^a^	0.995 ^ab^	15.61 ^c^	105.21 ^a^	98.84 ^e^	62.52 ^bc^
Succesiv	0.247 ^a^	1.170 ^ab^	11.76 ^e^	70.91 ^e^	119.31 ^c^	52.90 ^c^
Olivia	0.237 ^a^	1.031 ^ab^	11.65 ^e^	80.29 ^d^	91.59 ^f^	90.35 ^a^
SEM	0.009	0.133	0.063	0.735	0.719	1.014
R^2^	0.918	0.824	0.998	0.993	0.996	0.989
*p* value	0.082	0.049	<0.0001	<0.0001	<0.0001	0.022

Different letters (a, b, c, d, e, f) within each column indicate significant difference at *p* < 0.05 between seed varieties; Ca—calcium, P—phosphorus, Cu—copper, Fe—iron, Mn—manganese, Zn—zinc; SEM—standard error of the mean; R^2^—coefficient of determination of the ANOVA model; *p*—significance level according to ANOVA test; n = 3 biological replicates per cultivar.

**Table 3:** The fatty acid profiles of *Cannabis sativa* seeds.

Fatty Acids	Carbon Bond	Dacia Secuieni	Secuieni Jubileu	Ratza	Zenit	Succesiv	Olivia	SEM	R^2^	*p*
		g/100 g FAME								
Caprylic	C8:0	0.012 ^a^	0.008 ^a^	0.013 ^a^	0.012 ^a^	0.013 ^a^	0.011 ^a^	0.004	0.114	0.898
Capric	C10:0	0.015 ^a^	0.014 ^a^	0.017 ^a^	0.015 ^a^	0.013 ^a^	0.015 ^a^	0.003	0.088	0.941
Lauric	C12:0	0.012 ^a^	0.015 ^a^	0.017 ^a^	0.012 ^a^	0.016 ^a^	0.011 ^a^	0.003	0.175	0.764
Pentadecanoic	C15:0	0.015 ^a^	0.017 ^a^	0.013 ^a^	0.018 ^a^	0.016 ^a^	0.015 ^a^	0.004	0.064	0.971
Palmitic	C16:0	6.119 ^d^	6.682 ^bc^	6.977 ^a^	6.704 ^bc^	6.599 ^c^	6.852 ^ab^	0.053	0.941	<0.0001
Palmitoleic	C16:1	0.118 ^a^	0.116 ^a^	0.132 ^a^	0.124 ^a^	0.158 ^a^	0.130 ^a^	0.012	0.398	0.237
Heptadecenoic	C17:1	0.036 ^b^	0.044 ^ab^	0.070 ^a^	0.049 ^ab^	0.025 ^b^	0.057 ^ab^	0.008	0.671	0.011
Stearic	C18:0	2.693 ^b^	2.823 ^a^	2.741 ^ab^	2.373 ^d^	2.455 ^cd^	2.553 ^c^	0.025	0.957	<0.0001
Oleic	C18:1n-9	13.48 ^c^	14.31 ^b^	16.47 ^a^	13.15 ^d^	11.84 ^e^	13.16 ^d^	0.039	0.997	<0.0001
Linoleic (LA)	C18:2n6	54.27 ^c^	54.51 ^bc^	54.87 ^b^	54.87 ^b^	56.11 ^a^	56.16 ^a^	0.151	0.963	<0.0001
Linolenic γ (GLA)	C18:3n6	2.055 ^e^	3.551 ^c^	1.930 ^e^	4.307 ^a^	4.116 ^b^	2.877 ^d^	0.028	0.998	<0.0001
Linolenic α (ALA)	C18:3n3	19.76 ^a^	16.18 ^b^	15.20 ^c^	16.10 ^b^	16.35 ^b^	16.23 ^b^	0.107	0.982	<0.0001
Stearidonic (SDA)	C18:4n3	0.530 ^c^	0.800 ^b^	0.423 ^d^	1.097 ^a^	1.040 ^a^	0.747 ^b^	0.133	0.989	<0.0001
Arachidic	C20:0	0.012 ^a^	0.012 ^a^	0.013 ^a^	0.020 ^a^	0.016 ^a^	0.015 ^a^	0.004	0.153	0.816
Eicosadienoic	C20:2n6	0.051 ^a^	0.111 ^a^	0.060 ^a^	0.061 ^a^	0.204 ^a^	0.061 ^a^	0.061	0.282	0.488
Eicosatrienoic	C20:3n6	0.188 ^b^	0.237 ^a^	0.196 ^b^	0.211 ^ab^	0.218 ^ab^	0.219 ^ab^	0.007	0.667	0.012
Docosapentaenoic	C22:5n3	0.062 ^a^	0.077 ^a^	0.060 ^a^	0.054 ^a^	0.066 ^a^	0.057 ^a^	0.004	0.388	0.255
Other fatty acids		0.57 ^ab^	0.48 ^b^	0.80 ^a^	0.82 ^a^	0.74 ^ab^	0.83 ^a^	0.011	0.727	0.004
SFAs		8.88 ^d^	9.57 ^ab^	9.79 ^a^	9.15 ^c^	9.13 ^cd^	9.47 ^b^	0.021	0.937	<0.0001
MUFAs		13.63 ^c^	14.47 ^b^	16.674 ^a^	13.33 ^d^	12.03 ^e^	13.35 ^d^	0.018	0.998	<0.0001
PUFAs		76.92 ^b^	75.47 ^d^	72.74 ^e^	76.70 ^bc^	78.10 ^a^	76.35 ^c^	0.019	0.994	<0.0001
omega 3		20.35 ^a^	17.06 ^b^	15.68 ^c^	17.25 ^b^	17.45 ^b^	17.04 ^b^	0.261	0.980	<0.0001
omega 6		56.57 ^d^	58.41 ^c^	57.06 ^d^	59.45 ^b^	60.65 ^a^	59.32 ^b^	0.014	0.985	<0.0001
omega6/omega3		2.78 ^c^	3.43 ^b^	3.64 ^a^	3.45 ^b^	3.48 ^b^	3.48 ^b^	0.012	0.971	<0.0001

Different letters (a, b, c, d, e) within each row indicate significant difference at *p* < 0.05 between seeds varieties; SFAs—saturated fatty acids; MUFAs—monounsaturated fatty acids; PUFAs—polyunsaturated fatty acids; SEM—standard error of the mean; R^2^—coefficient of determination of the ANOVA model; *p*—significance level according to ANOVA test; n = 3 biological replicates per cultivar.

**Table 4:** Liposoluble antioxidant compounds of *Cannabis sativa* seeds.

Parameters	Total Tocopherol Content Determined				Carotenoids		
	α-Tocopherol	δ-Tocopherol	γ-Tocopherol	Total Tocopherol	Lutein	Astaxanthin *	Canthaxanthin *
	mg/kg	mg/kg	mg/kg	mg/kg	mg/kg	mg/kg	mg/kg
Dacia Secuieni	28.29 ^b^	23.37 ^a^	175.78 ^a^	227.44 ^a^	55.74 ^d^	1.30 ^a^	1.02 ^a^
Secuieni Jubileu	31.14 ^a^	18.39 ^c^	172.69 ^ab^	222.22 ^b^	47.73 ^e^	0.91 ^c^	0.75 ^b^
Ratza	28.62 ^b^	21.61 ^ab^	163.63 ^c^	213.86 ^c^	65.85 ^b^	1.29 ^a^	1.10 ^a^
Zenit	24.83 ^c^	14.82 ^d^	148.31 ^d^	187.96 ^d^	61.25 ^c^	1.32 ^a^	0.36 ^c^
Succesiv	21.94 ^d^	19.70 ^bc^	170.99 ^b^	212.63 ^c^	79.46 ^a^	1.17 ^b^	1.03 ^a^
Olivia	22.96 ^cd^	18.06 ^c^	130.35 ^e^	171.37 ^e^	55.12 ^d^	1.19 ^b^	0.19 ^d^
SEM	0.416	0.443	0.762	0.832	0.403	0.009	0.018
R^2^	0.969	0.950	0.996	0.996	0.997	0.991	0.995
*p* value	<0.0001	<0.0001	<0.0001	<0.0001	<0.0001	<0.0001	<0.0001

Different letters (a, b, c, d, e) within each column indicate significant difference at *p* < 0.05 between seed cultivars; SEM—standard error of the mean; R^2^—coefficient of determination of the ANOVA model; *p*—significance level according to ANOVA test; n = 3 biological replicates per cultivar. * Astaxanthin and canthaxanthin were tentatively assigned based on retention-time matching with the corresponding analytical standards and UV detection at 450 nm, without LC-MS/MS confirmation.

**Table 5:** Hydrosoluble antioxidant compounds of *Cannabis sativa* seeds.

Parameters	TPC,	DPPH,	TAC,	ICP,	ABTS,
	mg GAE/g	mmol TE/kg	mmol AAE/kg	mg EDTA/g	mmol TE/kg
Dacia Secuieni	1.27 ^ab^	12.80 ^a^	42.23 ^d^	2.70 ^b^	11.36 ^b^
Secuieni Jubileu	0.74 ^b^	12.54 ^a^	45.19 ^b^	2.42 ^d^	11.16 ^b^
Ratza	1.08 ^ab^	9.82 ^b^	43.89 ^c^	2.60 ^c^	9.25 ^b^
Zenit	1.50 ^a^	10.40 ^b^	44.95 ^b^	2.74 ^b^	14.97 ^a^
Succesiv	1.39 ^ab^	12.89 ^a^	43.48 ^c^	3.15 ^a^	11.15 ^b^
Olivia	1.19 ^ab^	7.91 ^c^	49.70 ^a^	2.22 ^e^	9.34 ^b^
SEM	0.140	0.230	0.097	0.012	0.586
R^2^	0.59	0.970	0.997	0.996	0.839
*p* value	0.040	<0.0001	<0.0001	<0.0001	<0.001

Different letters (a, b, c, d, e) within each column indicate significant difference at *p* < 0.05 between seed cultivars; SEM—standard error of the mean; R^2^—coefficient of determination of the ANOVA model; *p*—significance level according to ANOVA test; TPC—total polyphenol content; DPPH—2,2-diphenyl-1-picrylhydrazyl radical-scavenging activity; TAC—total antioxidant capacity (phosphomolybdenum method); ICP—iron chelation power; ABTS—2,2′-azino-bis(3-ethylbenzothiazoline-6-sulfonic acid); GAE—gallic acid equivalent; EDTA—ethylenediaminetetraacetic acid; n = 3 biological replicates per cultivar.

**Table 6:** Eigenvalues, variance, factor loadings, and scores from PCA.

Variable	F1	F2	F3
**PCA variances**			
Eigenvalue	5.34	3.31	2.69
Variance, %	36.00	22.40	18.14
Cumulative, %	36.00	58.40	76.44
**PCA loadings**			
Crude protein	−0.752	−0.483	−0.288
Crude fat	−0.726	−0.355	0.599
Crude fiber	0.696	−0.158	0.216
Iron	−0.526	−0.202	0.811
Manganese	−0.806	0.526	−0.334
Zinc	−0.066	−0.773	−0.264
Total tocopherols	−0.730	0.548	−0.282
Lutein	0.624	0.462	−0.358
Total polyphenol content	0.447	0.266	0.111
DPPH	−0.479	0.869	−0.144
ABTS	0.089	0.507	0.807
LA	0.885	−0.077	−0.432
GLA	0.568	0.592	0.468
ALA	−0.607	0.244	−0.250
**PCA scores**			
Dacia Secuieni	−3.437	0.504	−0.894
Secuieni Jubileu	−2.267	0.597	0.769
Ratza	−0.369	−1.708	−0.461
Zenit	1.440	0.449	3.138
Succesiv	2.557	2.773	−1.688
Olivia	2.075	−2.615	−0.864

PCA—principal component analyses; F1, F2, and F3 represent the first, second and the third principal components, respectively, accounting for 36.00%, 22.40% and 18.14% of the total variance; DPPH, 2,2-diphenyl-1-picrylhydrazyl radical-scavenging activity; ABTS, 2,2′-azino-bis(3-ethylbenzothiazoline-6-sulfonic acid) radical-scavenging activity; LA, linoleic acid (C18:2n-6); GLA, γ-linolenic acid (C18:3n-6); ALA, α-linolenic acid (C18:3n-3).

## Data Availability

The original contributions presented in the study are included in the article; further inquiries can be directed to the corresponding authors.
